# Supplementary material for: Electron confinement-enhanced green InP-based quantum dots for active-matrix LEDs displays
Source: Nat Commun. 2026 Feb 27;17:3268. doi: 10.1038/s41467-026-69050-7 (PMC13066630; doi:10.1038/s41467-026-69050-7)
Supplement: Supplementary file 1 — Supplementary Information [file 41467_2026_69050_MOESM1_ESM.pdf]

Supporting Information

Electron confinement-enhanced green InP-based quantum dots for active-matrix LEDs displays

Ning Guo[1,2,3†], Ke He[1,2,3†], Hui Li[4*], Tianchen Li[1,3], Fengmian Li[1], Jiangang Feng[4], Zhiyuan He[5*], Lei Jiang[1,4] and Yuchen Wu[1,2,3*]

[1]Key Laboratory of Bio-inspired Materials and Interfacial Science, Technical Institute of Physics and Chemistry, Chinese Academy of Sciences, Beijing 100190, P. R. China

[2]College of Chemistry, Jilin University, Changchun, 130012, P. R. China

[3]School of Future Technology, University of Chinese Academy of Sciences (UCAS) Beijing 100049, P. R. China

[4]State Key Laboratory of Bioinspired Interfacial Materials Science, Suzhou Institute for Advanced Research, University of Science and Technology of China, Suzhou, Jiangsu 215123, P. R. China

[5]School of Materials Science and Engineering, Beijing Institute of Technology, Beijing 100081, P. R. China

†These authors contributed equally.

*Corresponding authors. E-mails: lihui-t22031@ustc.edu.cn; hezy@bit.edu.cn; wuyuchen@iccas.ac.cn;

Supplementary Figures

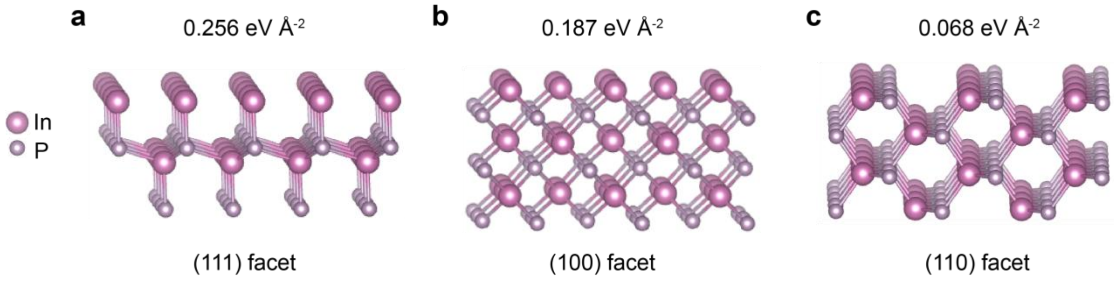

Supplementary Fig. 1 | DFT absolute surface energies of the three facets of InP. DFT absolute surface energies of the bare (**a**) (111) facet, (**b**) (100) facet and (**c**) (110) facet of lnP obtained from the slab models.

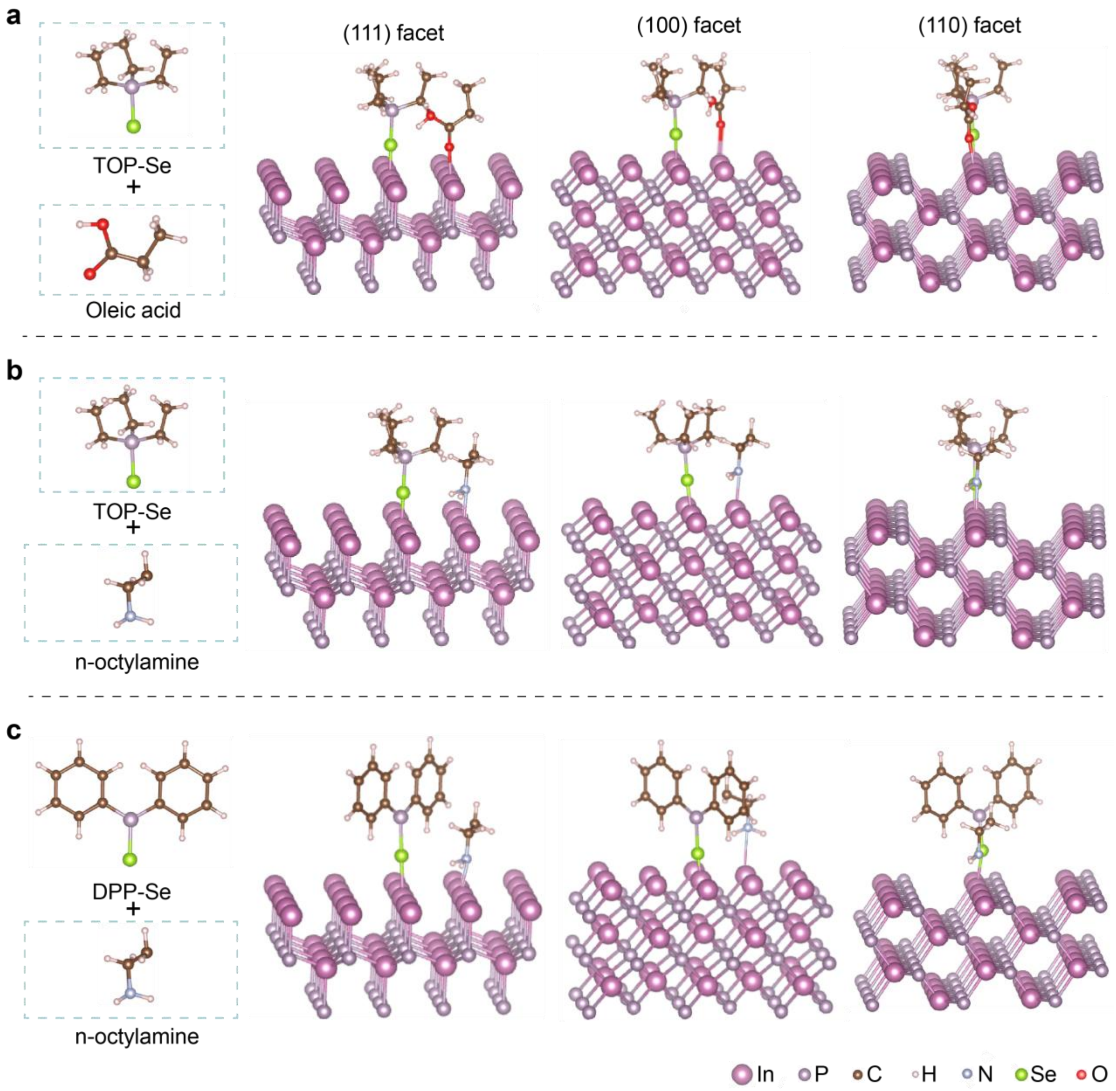

Supplementary Fig. 2 | Side views of the models with three different combinations of ligands coordinated on the three facets of InP. Models of three facets with (**a**) OA and TOP-Se, (**b**) n-octylamine and TOP-Se and (**c**) n-octylamine and DPP-Se.

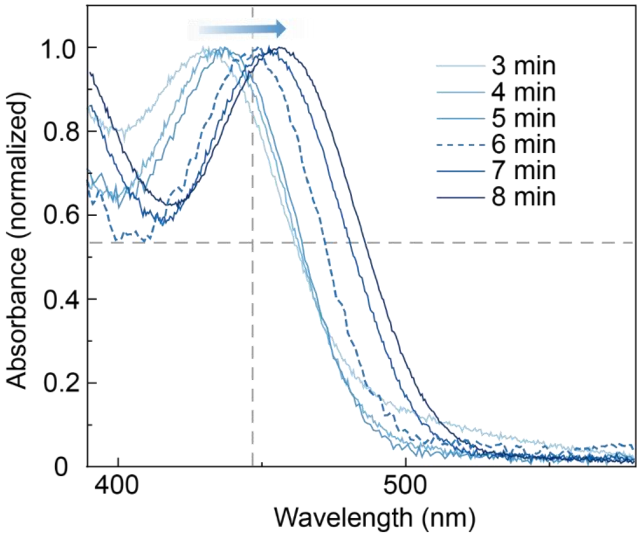

Supplementary Fig. 3 | Normalized UV-vis absorption spectra of the aliquots taken during the InP core synthesis. The InP core at the 6th minute exhibit the highest V/D value and was selected for shell growth process.

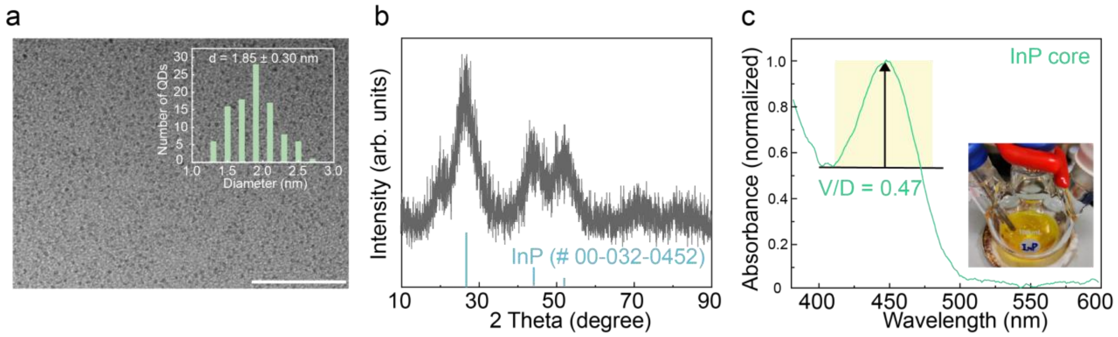

Supplementary Fig. 4 | Morphological and crystallographic characterization of InP cores. a, TEM images and particle size histogram of green InP core (average size: 1.85 ± 0.30 nm). Scale bar, 50 nm. **b,** XRD pattern of green InP core. **c,** UV-vis absorption spectrum of green InP core.

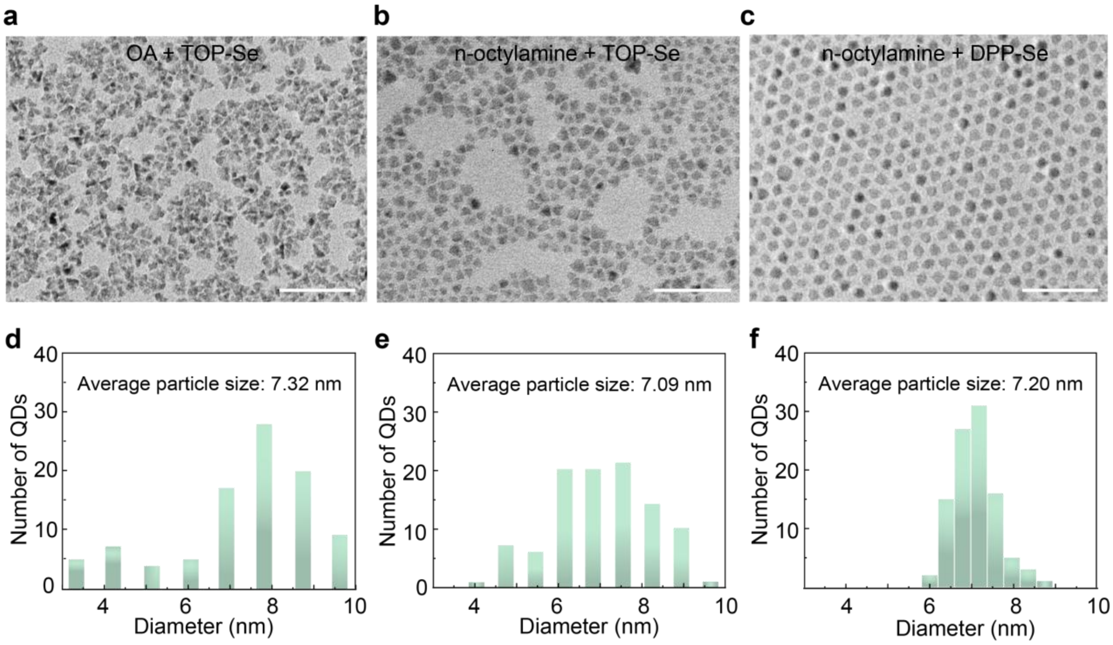

Supplementary Fig. 5 | Morphological characterization of QDs synthesized with three combinations of ligands. (**a-c**) TEM images and (**d-f**) corresponding histograms of the diameters of InP/ZnSe/ZnS QDs synthesized with three different ligand combinations of OA + TOP-Se, n-octylamine + TOP-Se and n-octylamine + DPP-Se. Scale bar, 50 nm.

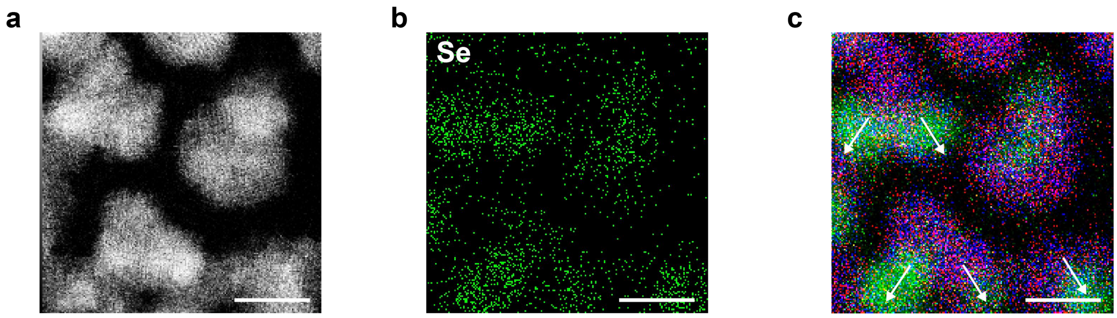

Supplementary Fig. 6 | Morphology of QDs synthesized with the combination of OA and TOP-Se. a, A high-angle annular dark field image. **b,** EDS mapping of Se elements. **c,** EDS mapping of Se (green), Zn (blue) and S (red) elements. Scale bar, 5 nm.

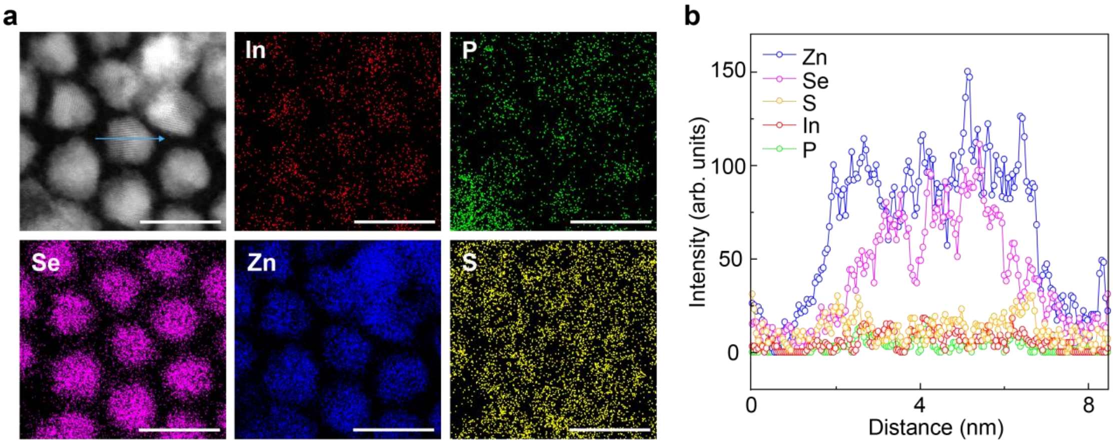

Supplementary Fig. 7 | Morphology of QDs synthesized with the combination of n-octylamine and DPP-Se. a, TEM image and electronic dispersive spectroscopy (EDS) mapping of In, P, Se, Zn, S of InP/ZnSe/ZnS QDs synthesized with the combination of n-octylamine + DPP-Se. Scale bar, 10 nm. **b**, Line profiles showing the distribution of elements of In, P, Se, Zn and S.

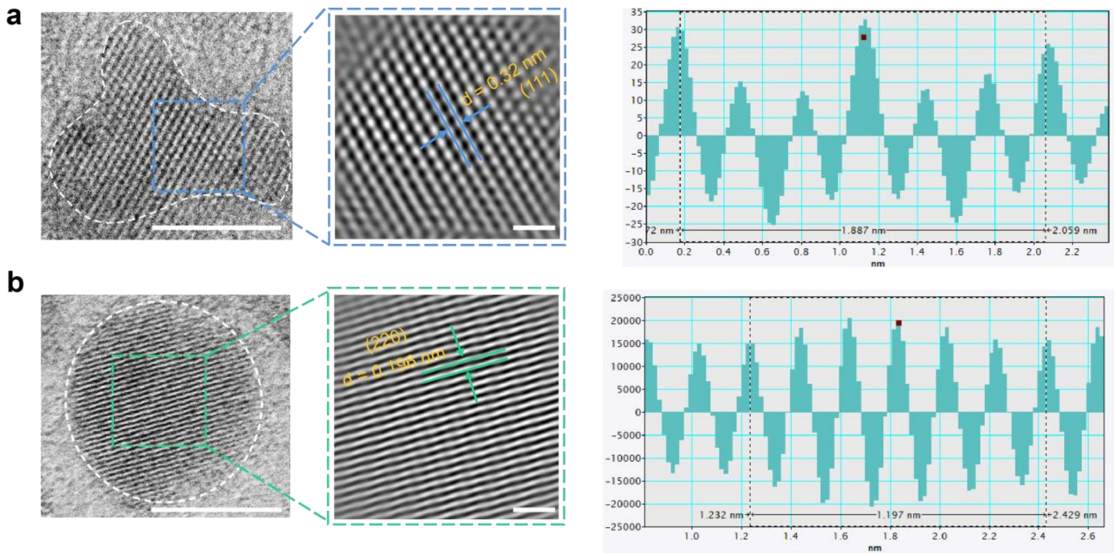

Supplementary Fig. 8 | HRTEM images and inverse Fourier transform images of QDs synthesized with the combination of (**a**) OA and TOP-Se and (**b**) n-octylamine and DPP-Se. The scale bars in the HRTEM images and the FFT images are 5 nm and 1 nm, respectively.

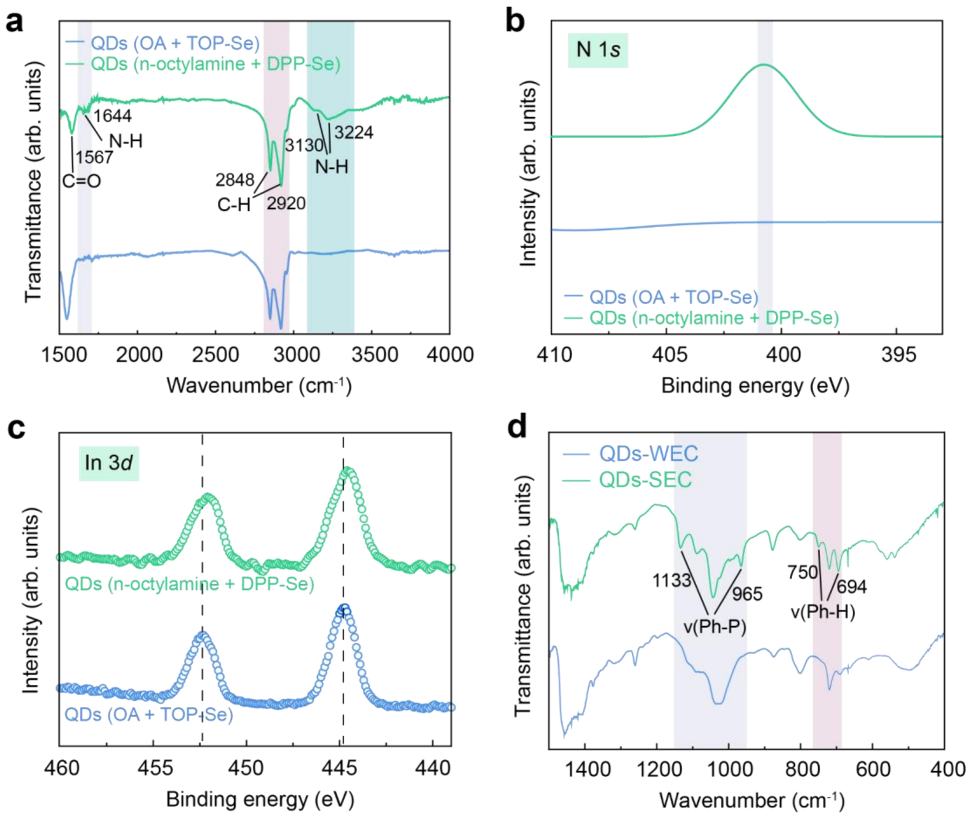

Supplementary Fig. 9 | Characterization of surface chemistry for two types of InP/ZnSe/ZnS QDs. a, Fourier-transform infrared spectra (FTIR) for two types of QDs characterizing the amino groups. **b,** High-resolution X-ray photoelectron spectroscopy spectra of N 1*s* for two types of QDs. **c,** High-resolution X-ray photoelectron spectroscopy spectra of In 3*d* for two types of QDs. **d,** FTIR for two types of QDs characterizing the DPP.

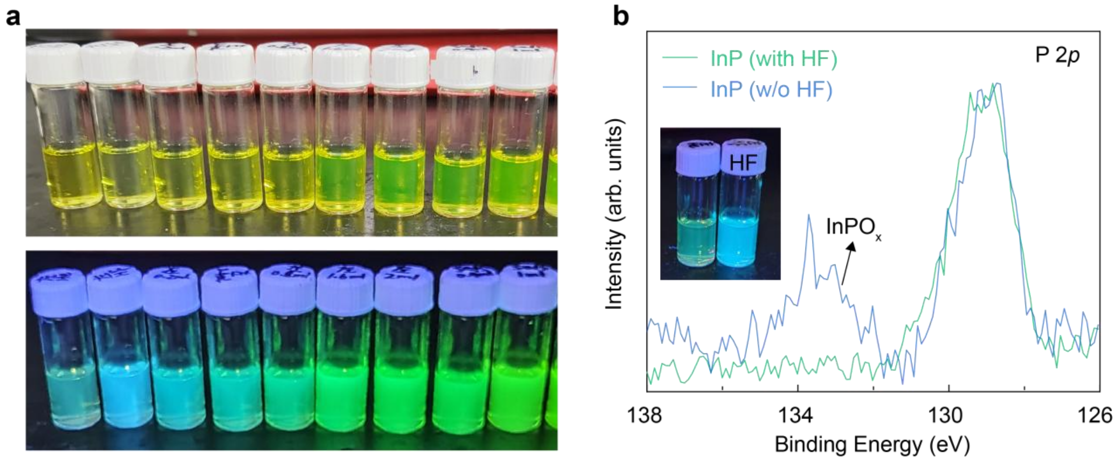

Supplementary Fig. 10 | Synthesis process of InP/ZnSe/ZnS QDs synthesized with n-octylamine + DPP-Se. a, Photographs of QD solution at different stages of synthesis process taken under natural light (top) and ultraviolet irradiation at 365 nm (bottom). **b,** High-resolution XPS signals of P 2*p* for InP cores before and after HF treatment.

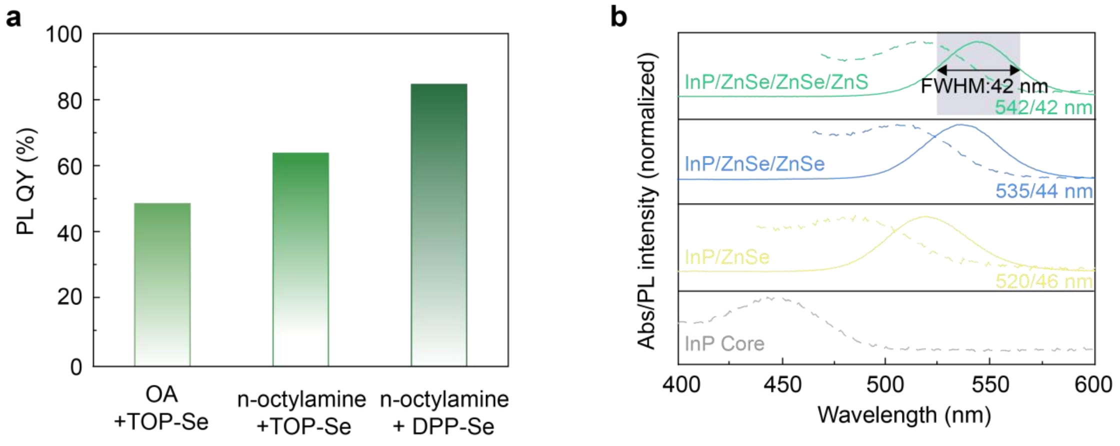

Supplementary Fig. 11 | PLQY and evolution of spectra during WEC QDs synthesis process. a, PLQY of InP/ZnSe QD synthesized with different ligand combinations. **b,** Evolution of absorption spectra (dashed line) and PL spectra (solid line) during WEC InP/ZnSe/ZnS QDs synthesis process.

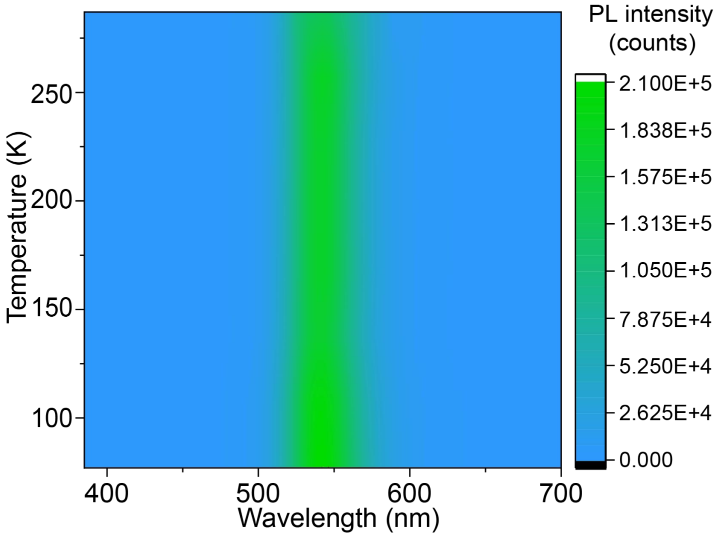

Supplementary Fig. 12 | Temperature-dependent PL spectra of WEC InP/ZnSe/ZnS QDs.

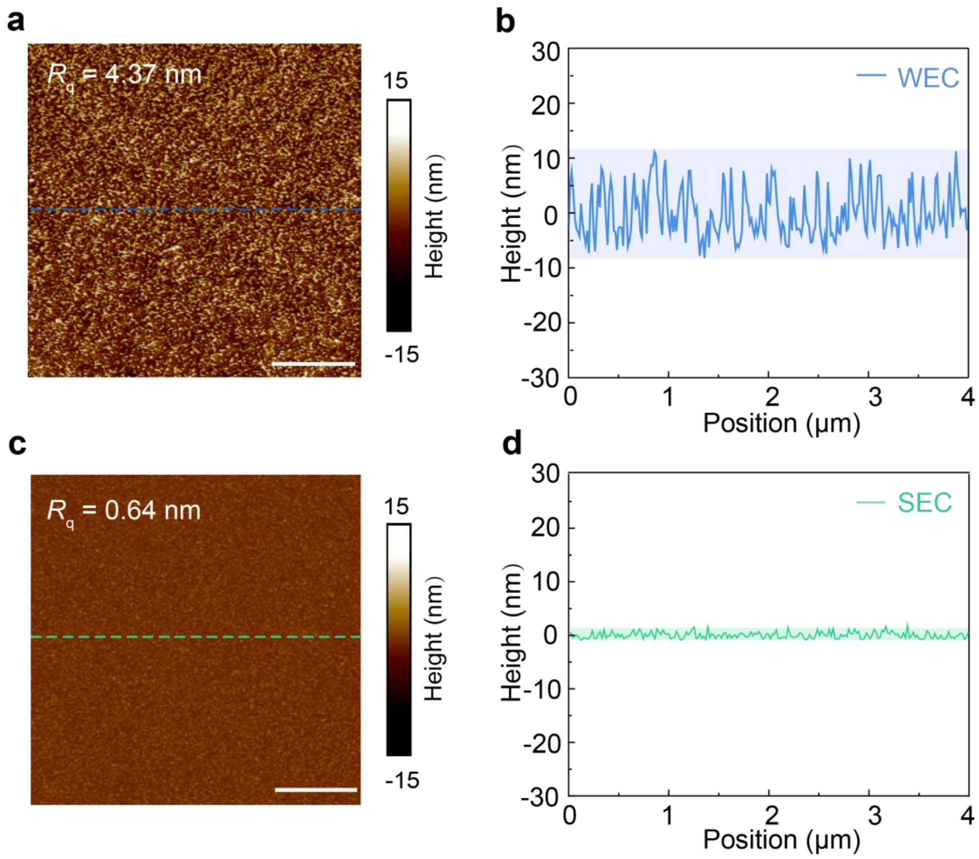

Supplementary Fig. 13 | Surface roughness for two types of QD thin films. a, AFM image of the WEC QD film. Scale bar, 1 μm. **b,** Corresponding height profile of the WEC QD film. **c,** AFM image of the SEC QD film. Scale bar, 1 μm. **d,** Corresponding height profile of the SEC QD film.

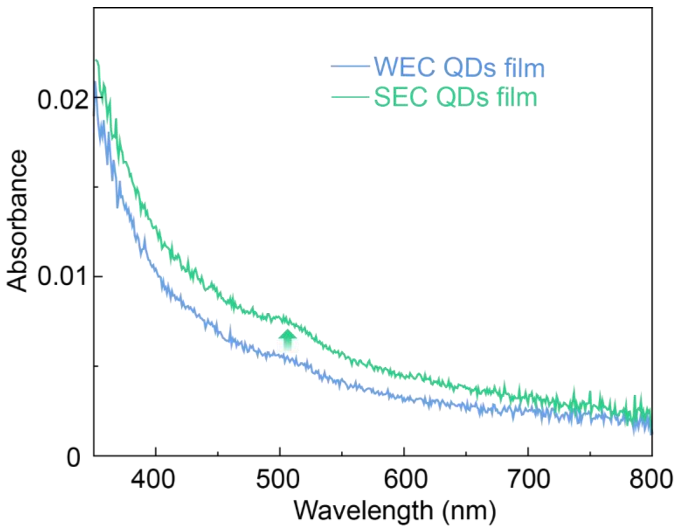

Supplementary Fig. 14 | UV-vis absorption spectra of two types of InP/ZnSe/ZnS QD film. The two films were both fabricated by spin-coating at a speed of 2,000 rpm.

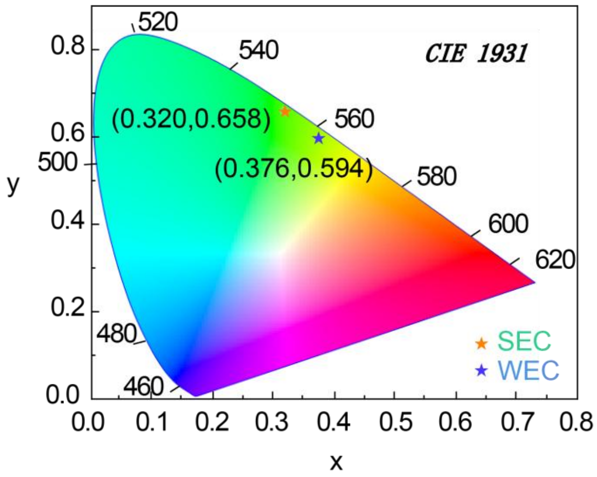

Supplementary Fig. 15 | Comparison of CIE diagram showing chromaticity coordinates of EL spectra of QLED devices based on SEC QD films and WEC QD films.

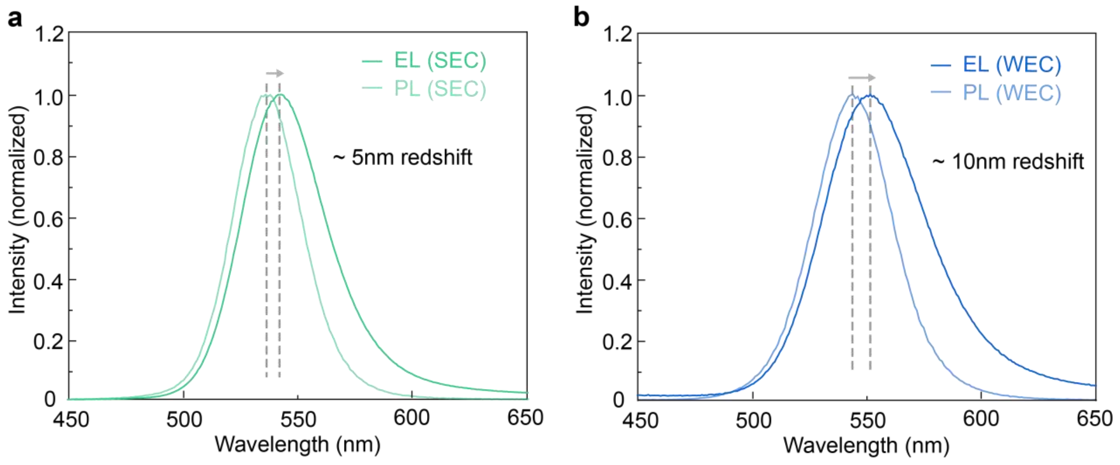

Supplementary Fig. 16 | A comparison between the EL and PL spectra of two types of QDs. a, EL spectra of QLED device based on SEC QD films exhibits a 5 nm redshift (537 nm to 542 nm) in peak positions compared to the PL spectra of the SEC QDs. **b,** EL spectra of QLED device based on WEC QD films exhibits a 10 nm (542 nm to 552 nm) redshift in peak positions compared to PL spectra of the WEC QDs.

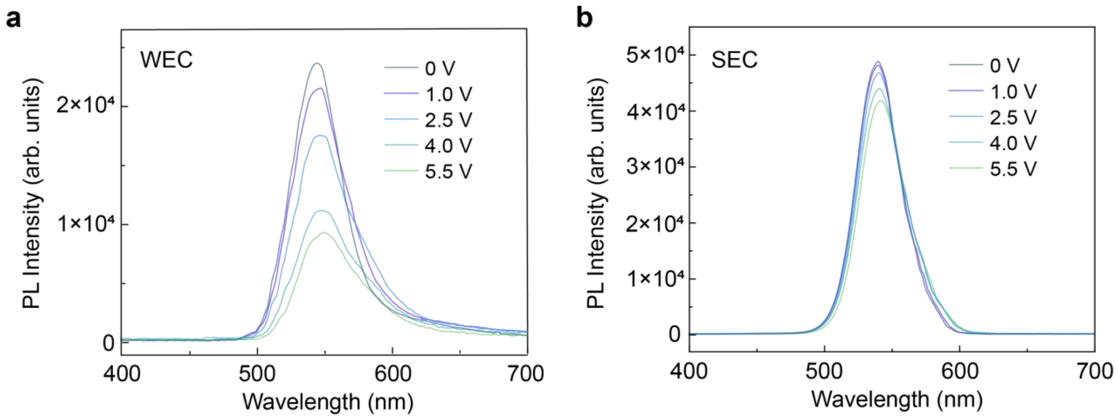

Supplementary Fig. 17 | PL spectra under different applied electric fields of (a) WEC QDs and (**b**) SEC QDs in EOD (ITO/ZnMgO/QD/ZnMgO/Al).

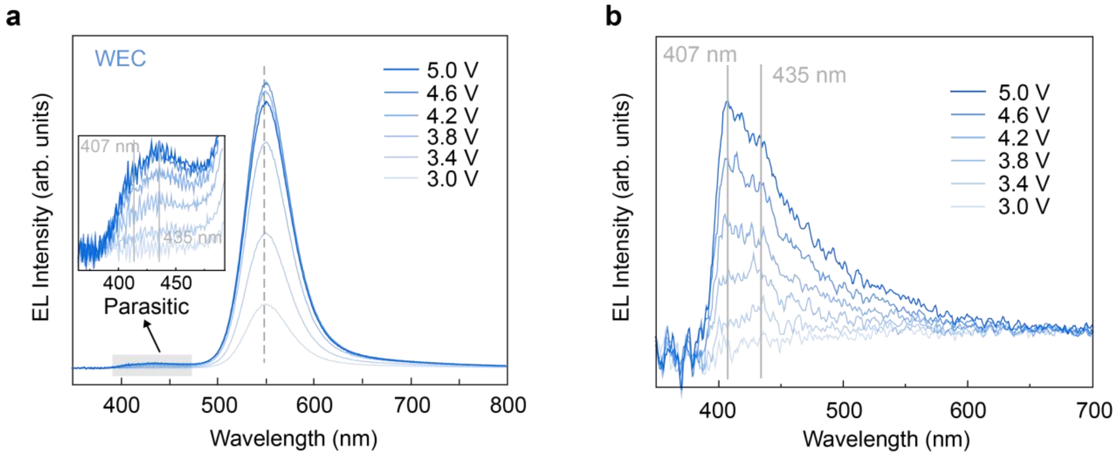

Supplementary Fig. 18 | HTL emission caused by highly delocalized electron leakage from the InP QD layer to the HTL, where they recombine with holes. a, EL spectra of QLED based on WEC InP/ZnSe/ZnS QD films under different voltages. (Inset: magnified spectra marked by the rectangle showing parasitic emission). **b,** EL spectrum of PF8Cz-based control device (with a structure of ITO/PEDOT:PSS/PF8Cz/ZnMgO/Al).

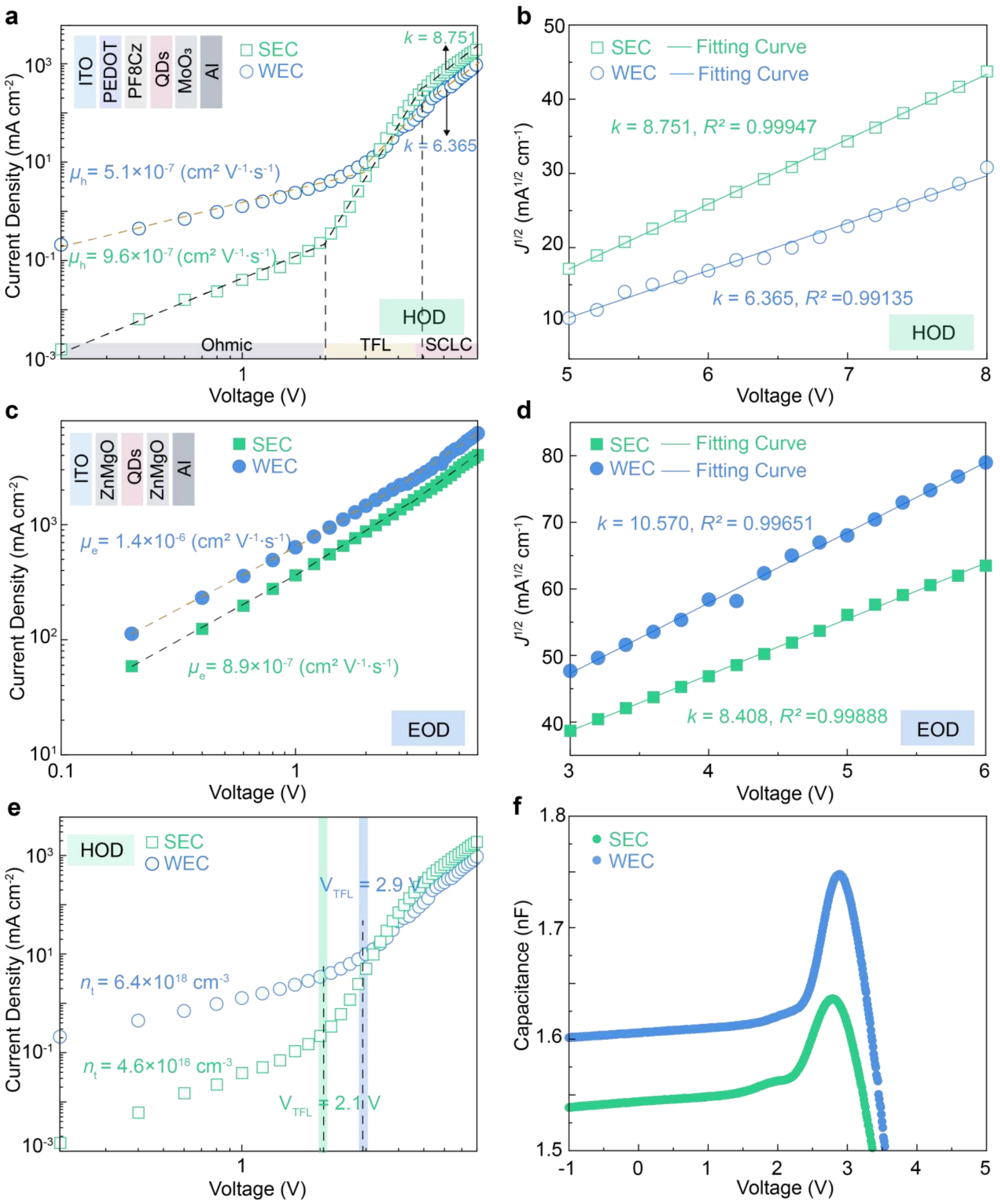

Supplementary Fig. 19 | Electrical properties of the QDs film based on SEC and WEC InP/ZnSe/ZnS QD. a, b, SCLC measurements of hole-only devices (HODs) based on SEC and WEC QD films. **c, d,** SCLC measurements of electron-only devices (EODs) based on SEC and WEC QD films. **e,** Defect state density (n_t) of the SEC and WEC QD films calculated from the V_{TFL} using the equation in Supplementary Note S6. f, The capacitance-voltage (C-V) characteristics of QLEDs based on SEC and WEC QD films.

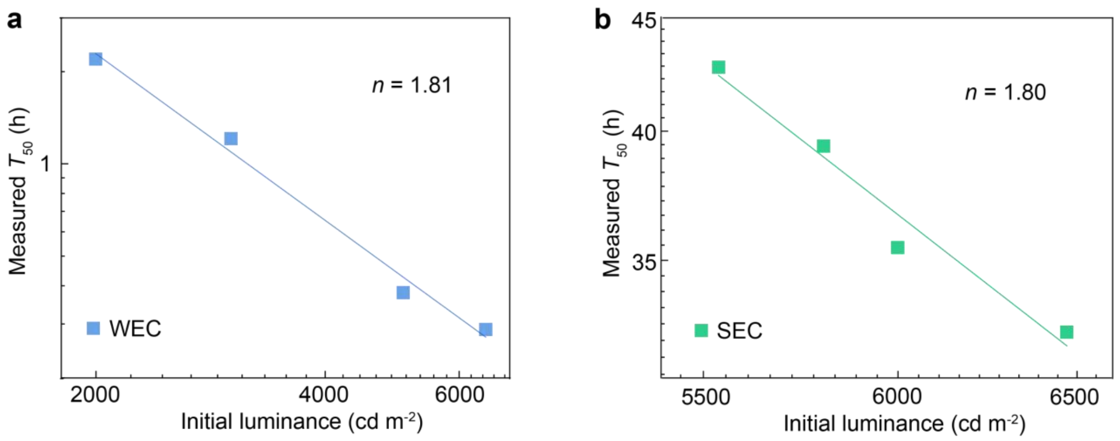

Supplementary Fig. 20 | Acceleration factor fittings for the QLEDs based on two types of QD films. The acceleration factors (n) for (**a**) QLEDs based on WEC QD films and (**b**) QLEDs based on SEC QD films were determined to be 1.81 and 1.80, respectively.

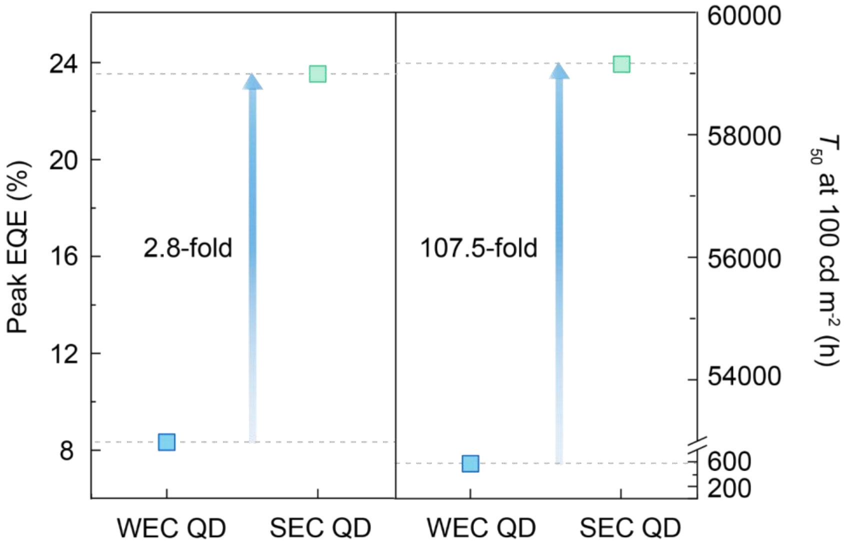

Supplementary Fig. 21 | Comparison of the peak EQE and calculated T_{50} at 100 cd m^{-2} between QLED devices based on SEC QD films and QLED devices based on WEC QD films.

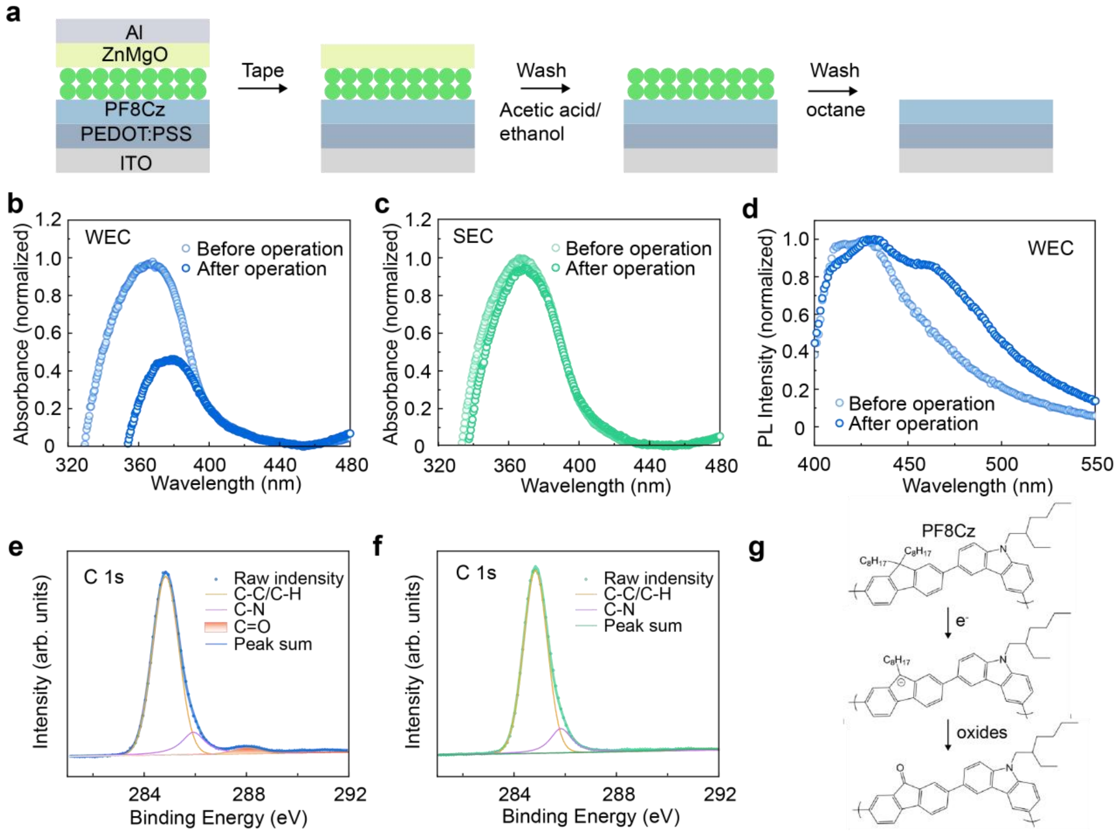

Supplementary Fig. 22 | The degradation mechanism of the HTL. a, Schematics of exposing the surfaces of PF8Cz in the QLEDs by removing all the top layers. **b, c,** Absorption spectra of PF8Cz HTLs in QLEDs based on (**b**) WEC QDs and (**c**) SEC QDs before and after operation at 2.0 mA cm^{-2} for 20 h. **d, e,** XPS spectra of PF8Cz HTLs in QLEDs based on (**d**) WEC QDs and (**e**) SEC QDs after electrical aging at 2.0 mA cm^{-2} for 20 h. **f,** PL spectra of PF8Cz in QLEDs based on WEC QDs before and after operation at 2.0 mA cm^{-2} for 20 h. **g,** The mechanism of electron leakage initiating HTL degradation by the generating fluorenone defect sites in PF8Cz.

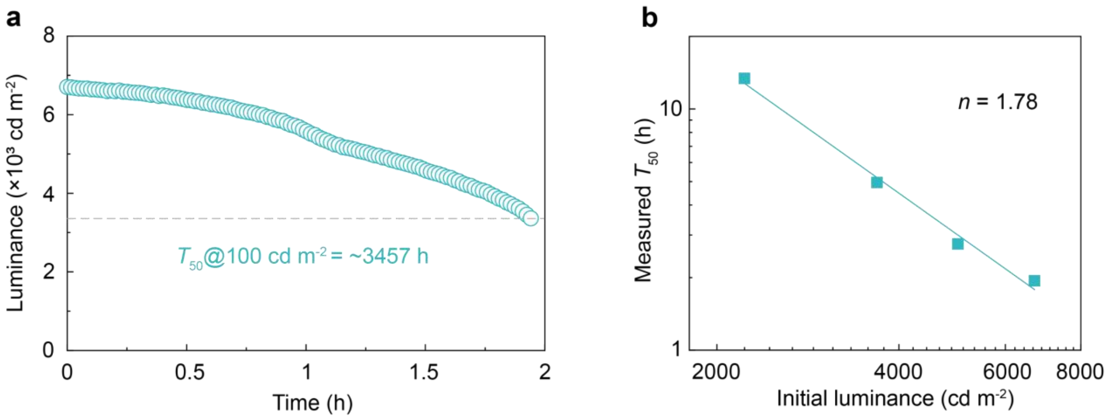

Supplementary Fig. 23 | Lifetime measurements of QLED devices based on InP/ZnSe/ZnS QDs synthesized using n-octylamine and DPP-Se with a thinner ZnSe shell (~1 nm). a, Luminance and time dependency characteristics curves of devices based on InP/ZnSe/ZnS QDs synthesized using n-octylamine and DPP-Se with a thinner ZnSe shell (~1 nm). **b,** Extrapolation of accelerating factor (*n*) for the lifetime estimation.

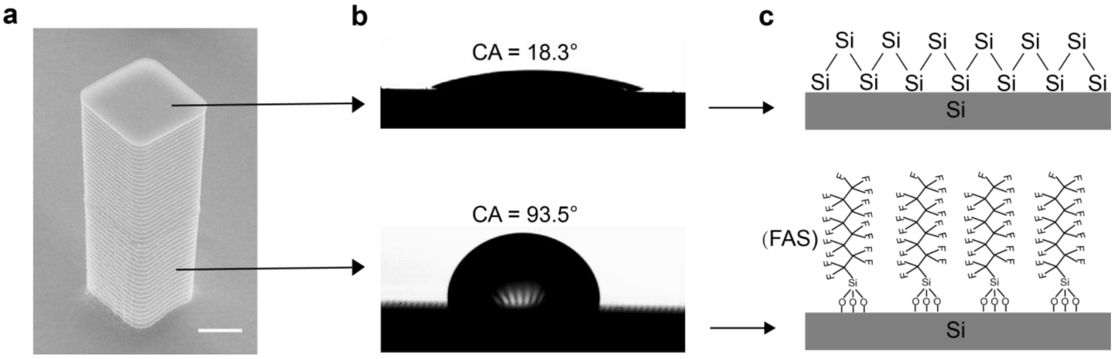

Supplementary Fig. 24 | Asymmetric wettability modification of the micropillar template. a, A SEM image of a microsquare pillar modified by FAS. Scale bar, 2 μm. **b,** The water contact angle (CA) of the top of the micropillar is 18.3° ± 0.5°, exhibiting the hydrophilicity. The sidewall of the micropillar shows hydrophobicity with the CA of 93.5° ± 0.5°. **c,** Schematic diagram of FAS modification on the sidewall of micropillars.

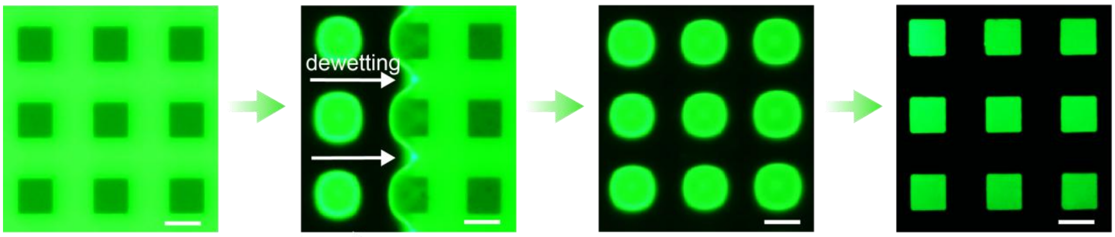

Supplementary Fig. 25 | In-situ fluorescence microscopy images of the dewetting process of QD solutions. QD solution underwent directional dewetting to assemble QD microstructure array. Scale bar, 10 μm.

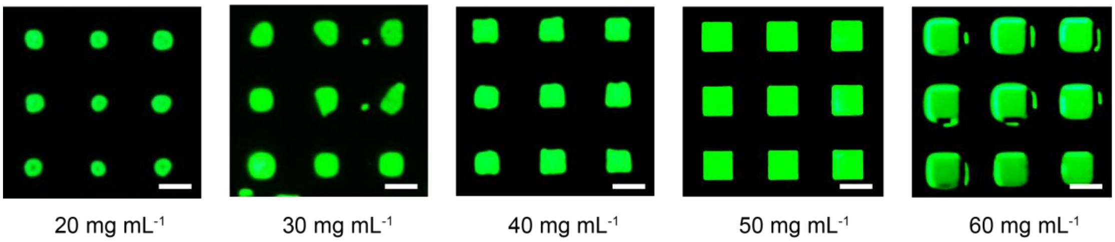

Supplementary Fig. 26 | The influence of QD solution concentration on the assembly morphology of the microstructure array. The fluorescence microscopy images of microstructure arrays assembled by QD solution of different concentration demonstrates that the assembled microstructure array exhibits the best morphology when the QD concentration is 50 mg mL^{-1}. Scale bar, 10 μm.

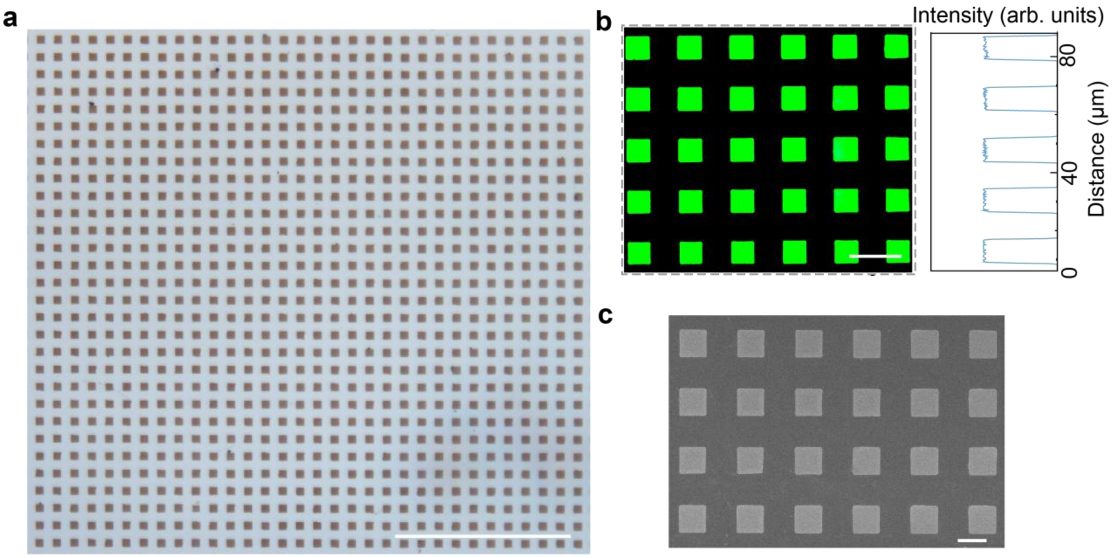

Supplementary Fig. 27 | Characterization of the QD microstructure array. a, Large area optical microscope image of the QD microstructure array. Scale bar, 200 μm. **b,** Fluorescence images of the QD microstructure array, which showing uniform PL intensity under confocal microscopy. Scale bar, 20 μm. **c,** Scanning electron microscopy image of the QD microstructure array. Scale bar, 10 μm.

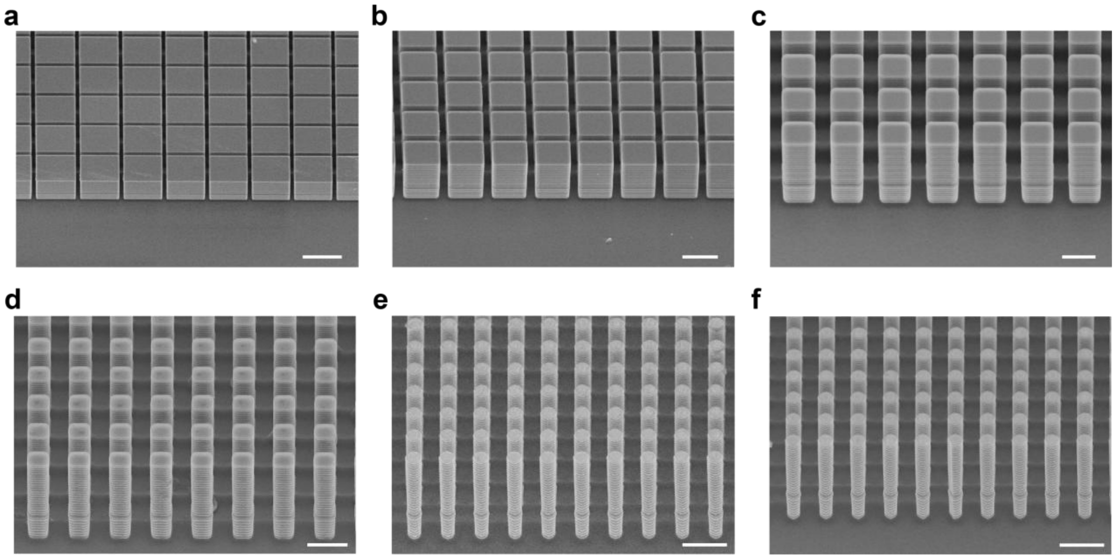

Supplementary Fig. 28 | Scanning electron microscopy images of micropillar templates with square micropillar of different sizes: (**a**) 20-2 μm, (**b**) 10-2 μm, (**c**) 5-2 μm, (**d**) 3-2 μm, (**e**) 2-2 μm, (**f**) 1.5-1.5 μm. (The preceding numerical value denotes the side length of the micropillars, while the subsequent value denotes the inter-pillar spacing between adjacent micropillars). Scale bars, 20 μm (**a**), 10 μm (**b**) and 5 μm (**c-f**).

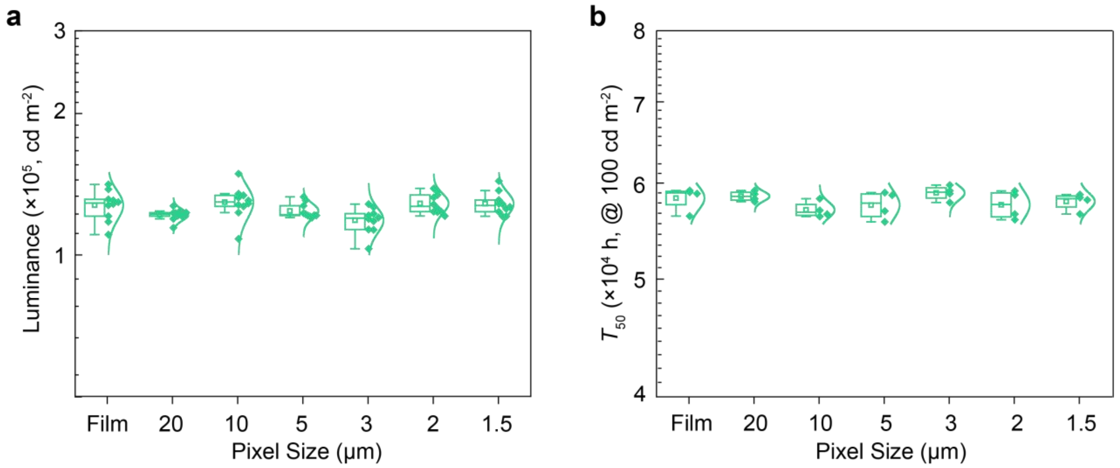

Supplementary Fig. 29 | Luminance and operational lifetime of high-resolution QLED devices. a, Statistics of maximum luminance of devices with characteristic pixel size. **b,** Statistics of operational lifetime (T_{50} @ 100 cd m^{-2}) of devices with characteristic pixel size. The solid diamonds correspond to the datas of 10 devices with corresponding pixel size. The curves are the Gaussian fitting to the data distributions. The box plots and Gaussian fitting were generated from the EQE data, indicating the mean (empty squares), standard deviation, lower quartile (25%), median (50%), upper quartile (75%), interquartile range (25–75%) and maximum/minimum (crosses) of the data. Error bars represent the standard deviation of 10 devices.

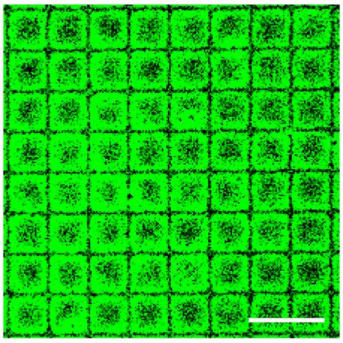

Supplementary Fig. 30 | Fluorescence micrograph of spin-coated micro-LEDs based on SEC QDs. Scale bar, 20 μm.

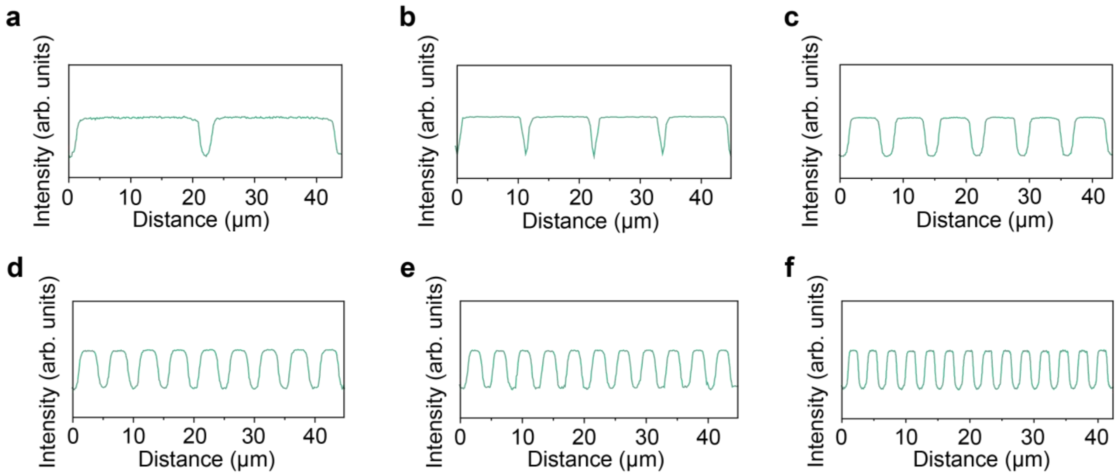

Supplementary Fig. 31 | The EL intensity linecut of high-resolution QLEDs with different pixel sizes under confocal microscopy. (**a**) 20-2 μm, (**b**) 10-2 μm, (**c**) 5-2 μm, (**d**) 3-2 μm, (**e**) 2-2 μm, (**f**) 1.5-1.5 μm. The former value represents the side length of the pixel, and the latter value represents the distance between adjacent pixels. Scale bar, 10 μm.

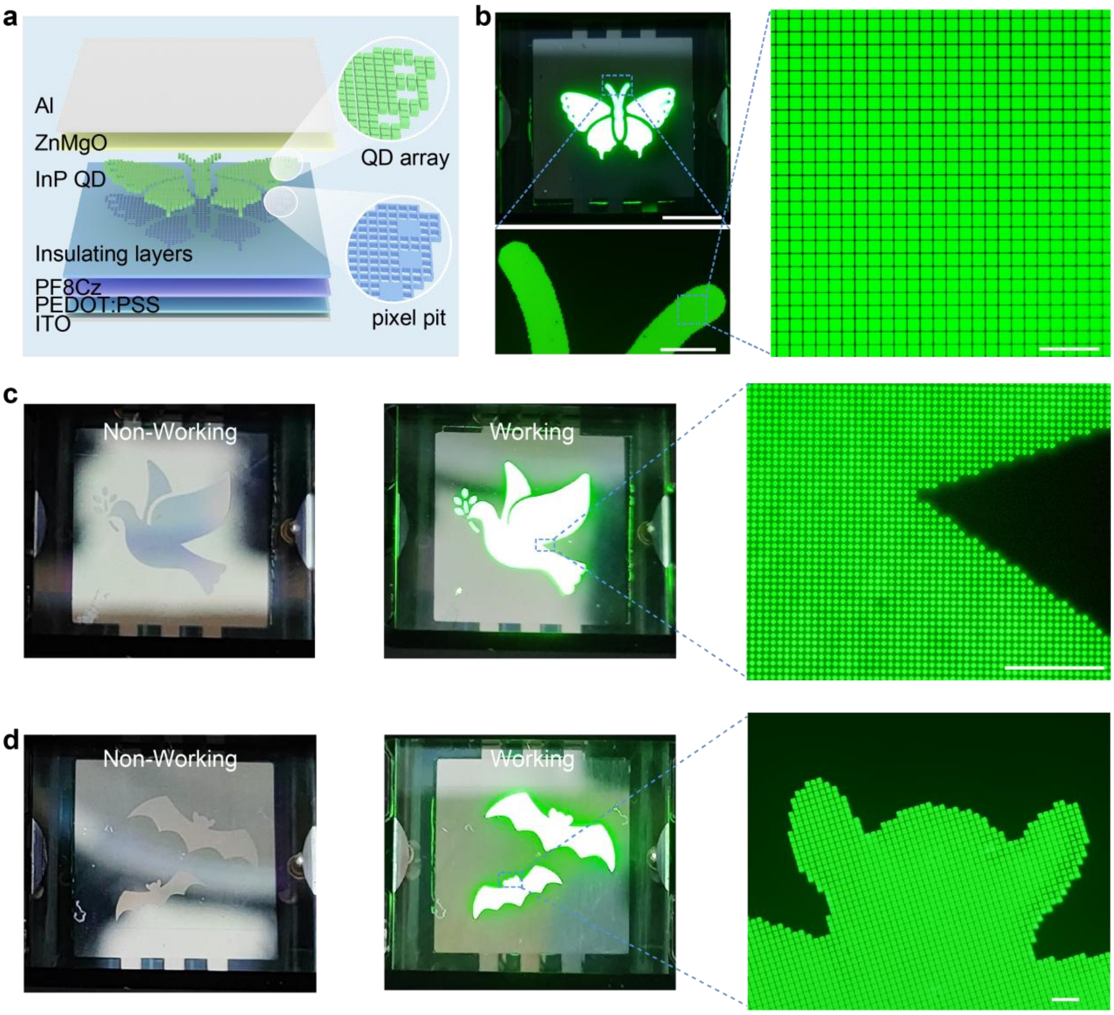

Supplementary Fig. 32 | Patterned high-resolution QLED. a, Schematic illustration of the structure of the patterned high-resolution QLED device, in which the QDs are within the pixel pits. **b,** Photograph of an operating patterned QLED device displaying a butterfly (top left) (Scale bar, 5 mm), the corresponding microscopy image (bottom left) (Scale bar, 500 μm) and the amplifying image (right) (Scale bar, 100 μm) showing the pixel array of the antenna of the butterfly. **c, d,** Photographs of the patterned QLED displaying (**c**) a dove with a pixel size of 5 μm and (**d**) two bats with a pixel size of 20 μm at non-working state (left), working state (middle) and microscopy image of the corresponding area (right). Scale bar, 100 μm.

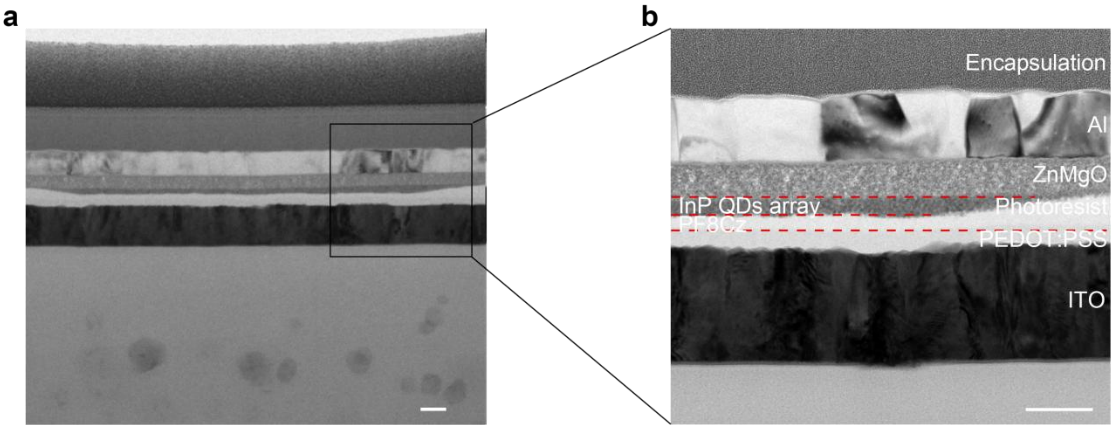

Supplementary Fig. 33 | Cross-section of the patterned high-resolution QLED. a, Cross-sectional TEM images of the QLED with patterned QD arrays. **b,** A magnified view on the patterned QLED structure. Scale bar, 100 nm.

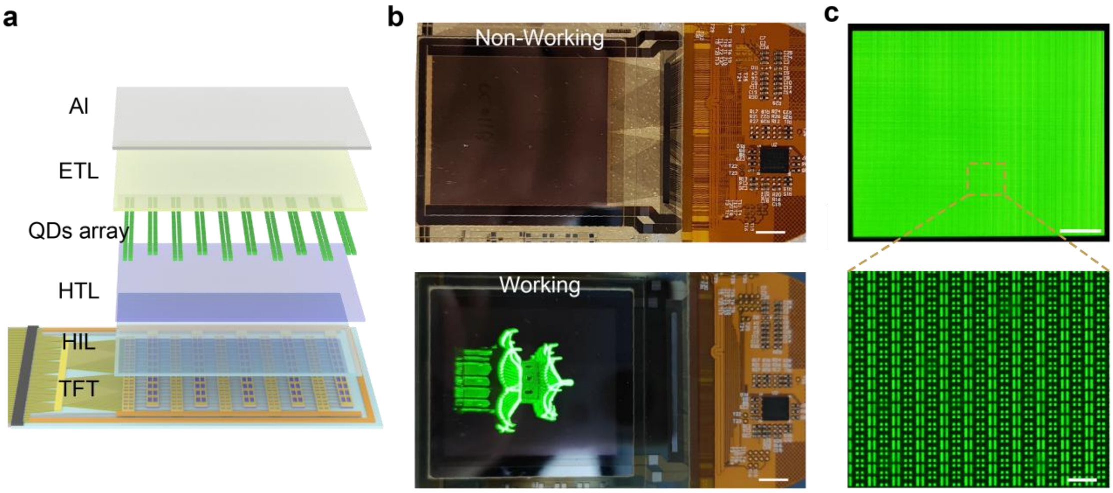

Supplementary Fig. 34 | Active-matrix LEDs displays. a, Schematic illustration of the structure of an active-matrix LEDs display. **b,** Photographs of the active-matrix LED display at non-working state (top) and working state (bottom). Scale bar, 10 mm. **c,** Photograph of the display with all the pixels at its luminance (top) and corresponding microscopy image (bottom) displaying the pixels. Scale bars, 10 mm (top) and 200 μm (bottom).

Supplementary Tables

Supplementary Table 1 | DFT formation energies of the three ligand combinations on (100), (110) and (111) facets of lnP obtained from the wedge and slab models.

Facets	Ligands	Post-adsorption energy (eV)	Post-adsorption surface energy (eV Å^{-2})	Formation energy (eV)
(111)	OA+TOP-Se	-413.62	0.1507	-25.77
	n-octylamine +TOP-Se	-406.23	0.1111	-35.49
	n-octylamine +DPP-Se	-442.90	0.0968	-38.98
(100)	OA+TOP-Se	-433.96	0.1174	-9.91
	n-octylamine +TOP-Se	-421.53	0.0844	-14.58
	n-octylamine +DPP-Se	-454.61	0.0852	-14.48
(110)	OA+TOP-Se	-437.76	0.0641	-0.68
	n-octylamine +TOP-Se	-421.06	0.0620	-1.09
	n-octylamine +DPP-Se	-454.09	0.0628	-0.94

Supplementary Table 2 | Fitting parameters of the kinetic traces of the TA data of the two types of QDs at pump power of 420 μW.

	WEC QDs	SEC QDs
Source	513 nm	510 nm
A_1%	44.7	17.3
τ_1 (ps)	70.6	136
A_2%	29.4	10.2
τ_2 (ps)	20200	6180
A_3%	25.9	72.6
τ_3 (ps)	17600	20400

Supplementary Table 3 | Stability tests of two types of QDs. The table below presents the PL QY values for two types of QD solutions under continuous irradiation at 365 nm ultraviolet light.

Time (h)	PL QY of WEC QDs (%)	PL QY of SEC QDs (%)
0	64.46	92.28
5	58.08	91.91
10	49.02	88.66
15	46.56	84.39
20	40.08	80.91
25	22.93	77.39

Experimental section

Supplementary Note 1 | Precursor preparation for synthesis of InP/ZnSe/ZnS QDs

Materials

Zinc acetate ($Zn(OAc)_2$, 99.99%), sulfur (S, 99.998%, powder) oleic acid (OA, 90%) were purchased from Sigma-Aldrich. Selenium (Se, 99.999%, powder) and 1-octadecene (ODE, 90%) were purchased from Thermo scientific. Trioctylphosphine (TOP, 90%) and diphenylphosphine (DPP, ≥ 95%) were purchased from Aladdin.

Synthesis of the $Zn(OA)_2$ complex

In a nitrogen atmosphere, 20 mmol of $Zn(OAc)_2$ was mixed with 20 mL of OA and 30 mL of ODE within a flask. The resulting mixture was subjected to degassing at 140 °C for a duration of 30 min until the solution achieved a clear, transparent state, after which it was cooled to 100 °C and set aside for further use.

Synthesis of the TOP-S complex

Under an atmosphere of nitrogen in a glove box, accurately weigh 20 mmol of S powder and mix it with 20 mL of TOP. Stir the mixture thoroughly until the solution attains a clear, transparent state. The prepared TOP-S solution is 1.0 mmol mL^{-1}.

Synthesis of the TOP-Se complex

Under an atmosphere of nitrogen in a glove box, accurately weigh 10 mmol of Se powder and mix it with 25 mL of TOP. Stir the mixture thoroughly until the solution attains a clear, transparent state. The prepared TOP-Se solution is 0.4 mmol mL^{-1}.

Synthesis of the DPP-Se complex

Under an atmosphere of nitrogen in a glove box, accurately weigh 8 mmol of Se powder with 10 mL of DPP. Stir the mixture thoroughly and heat it slightly until the solution attains a clear, transparent state. The prepared DPP-Se solution is 0.4 mmol mL^{-1}.

Preparation of the 7 wt% HF solution

0.6 mL of hydrogen fluoride-pyridine was diluted with 6 mL of acetone.

Supplementary Note 2 | Synthesis of InP Core

Materials

Indium acetate ($In(OAc)_3$, 99.99%) and myristic acid (HMy, 98%) were purchased from Sigma-Aldrich. Tris(trimethylsilyl)-phosphine ($(TMS)_3P$, 20%) was purchased from Suzhou Xingshuo Nanotech Co., Ltd. 1-octadecene (ODE, 90%) was purchased from Thermo scientific. Trioctylphosphine (TOP, 90%), zinc undecylenate (≥ 98%) were purchased from Aladdin. Ethanol (99.7%) and toluene (≥ 99.5%) were purchased from Sinopharm Chemical Reagent Co., Ltd.

Synthesis of InP Core

A high-quality InP core was synthesized according to a previously reported method with some modifications. 0.2 mmol $In(OAc)_3$, 0.6 mmol HMy, 0.45 mmol zinc undecylenate and 10 mL ODE were mixed in a 100 mL flask. After a period of purging with nitrogen, it was degassed at 130 ℃ for 2 h under vacuum and back-filled with nitrogen. Subsequently, when the flask was heated up to 280 ℃, a complex of 0.15 mmol $(TMS)_3P$ diluted with 1 mL of TOP was injected into the reaction flask instantaneously. Temperature was maintained at 280 ℃ for 6 min to form InP core and cool it down to room temperature to cease the reaction. The product was then purified with ethanol and toluene for one time. Following this, the mixture was dispersed in 2 mL toluene in preparation for the growth of the shell.

Supplementary Note 3 | Synthesis of $Zn_{0.9}Mg_{0.1}O$ nanocrystals

Materials

Magnesium acetate tetrahydrate ($Mg(OAc)_2·4H_2O$, 99%) were purchased from Sigma-Aldrich. n-Hexanes (98%), tetramethylammonium hydroxide pentahydrate ($TMAH·5H_2O$, 97%), zinc acetate dihydrate ($Zn(OAc)_2·2H_2O$, 99.995%) and dimethyl sulfoxide (DMSO, 99.7%) were purchased from Aladdin. Ethanol (99.7%) was purchased from Sinopharm Chemical Reagent Co., Ltd.

Synthesis of $Zn_{0.9}Mg_{0.1}O$ nanocrystals

2.7 mmol of $Zn(OAc)_2·2H_2O$ and 0.3 mmol of $Mg(OAc)_2·4H_2O$ were dissolved in 30 mL DMSO (the mass fraction of $Mg(OAc)_2·4H_2O$ was 12.5%). Subsequently, 8.75 mL ethanol solution of 0.5 mmol mL^{-1} $TMAH·5H_2O$ was added to the precursor solution, followed by stirring for 2 h at 18-20 °C. Then the solution was centrifuged and washed twice with ethanol and n-hexanes. Finally, the precipitates were dispersed in ethanol to form a ZnMgO nanoparticle solution at a concentration of 20 mg mL^{-1}.

Supplementary Note 4 | Fabrication of HODs and EODs

Fabrication of HODs:

PEDOT:PSS was spin-coated onto ITO substrates at 4,000 rpm for 40 s, followed by annealing at 150 °C for 30 min. Then, these substrates were transferred into a nitrogen-filled glove box for spin-coating. PF8Cz with a concentration of 8 mg mL^{-1} (solvent: chlorobenzene) was spin-coating at 3,000 rpm for 40 s, and then baked at 120 °C for 30 min. The QD layer was prepared by spin-coating QD solution (20 mg mL^{-1}) at 2,000 rpm for 30 s and baked at 80 °C for 7 min. MoO_3 (with a thickness of 40 nm) and Al electrode (with a thickness of 100 nm) was successively deposited by thermal evaporation under a degree of vacuum of ≈ 2.5×10^{-4} Pa. Ultimately, the devices were encapsulated using UV-curable epoxy resin and cover glass in a glove box.

Fabrication of EODs:

ZnMgO nanoparticles solution (20 mg mL^{-1}) was spun-coating at 2,000 rpm for 30 s and baked at 60 °C for 30 min. Subsequently, QD layer was prepared by spin-coating QD solution (20 mg mL^{-1}) at 2,000 rpm for 30 s and baked at 80 °C for 7 min, followed by a second spin-coating of the ZnMgO layer in the same parameters as before. Then, Al electrode (with a thickness of 100 nm) was deposited by thermal evaporation under a degree of vacuum of ≈ 2.5×10^{-4} Pa. Ultimately, the devices were encapsulated using UV-curable epoxy resin and cover glass in a glove box.

Supplementary Note 5 | The calculation of carrier mobility and defect state density

Calculation of carrier mobility

The *I-V* behavior of the devices exhibits three regions, each defined by a slope k: the Ohmic regime ($k = 1$), the trap-filled limit (TFL) regime ($k > 3$), and the Child regime, which increases with applied bias ($k = 2$). By fitting the Child regime and integrating the fit using the Mott-Gurney equation, the mobility μ was obtained using the following equation[1]:

$$\mu = \frac{8JL^3}{9\varepsilon_0\varepsilon_r V^2} \tag{1}$$

where J is the dark current density measured in the SCLC region, V is the applied voltage, ε_0 is the vacuum permittivity, ε_r is the relative dielectric constant and L is the thickness of the QD film, respectively.

Calculation of defect state density

Defect state density (n_t) of the QLED device can be calculated from trap-filled limit voltage (V_{TFL})[2]:

$$n_t = \frac{2\varepsilon_0\varepsilon_r V_{TFL}}{eL^2} \tag{2}$$

where ε_0 is the vacuum permittivity, ε_r is the relative dielectric constant, V_{TFL} is the onset voltage of the TFL region, e is the electron charge and L is the thickness of the QD film, respectively.

Supplementary Note 6 | FAS modification of micropillar templates

Materials

Heptadecafluorodecyltrimethoxysilane (FAS, 98%) and acetone (98%) were purchased from Aladdin.

FAS modification of micropillar templates

A substrate coated with SU-8 photoresist is affixed to the top of the micropillars on the template to protect the top of the micropillars from being modified by FAS. Subsequently, the top-protected micropillar topography template is placed in a dryer, and 20 μL of FAS solution is added. The salinization with FAS is conducted for 1 h under reduced pressure at room temperature, after which the dryer is placed in an oven at 90°C for a duration of 2 h. Finally, the SU-8 photoresist is dissolved with acetone, resulting in asymmetric wettability on the top and sidewalls of each micropillar on the template.

Supplementary References

1. Wang, Y. et al. Ligand-Solvent Coordination Enables Comprehensive Trap Passivation for Efficient Near-Infrared Quantum Dot Light-Emitting Diodes. *Angew. Chem. Int. Ed.* **63**, e202407833 (2024).

2. Kong, L. et al. Fabrication of red-emitting perovskite LEDs by stabilizing their octahedral structure. *Nature* **631**, 73-79 (2024).
