# Supplementary material for: Electron confinement-enhanced green InP-based quantum dots for active-matrix LEDs displays
Source: Nat Commun. 2026 Feb 27;17:3268. doi: 10.1038/s41467-026-69050-7 (PMC13066630; doi:10.1038/s41467-026-69050-7)
Supplement: Supplementary file 2 — Description of Additional Supplementary Files [file 41467_2026_69050_MOESM2_ESM.pdf]

Description of Additional Supplementary Files

Supplementary Video 1. A vivid video of the active-matrix LEDs displays showing the flow of water droplets.
